# Autophagy-Associated Atrophy and Metabolic Remodeling of the Mouse Diaphragm after Short-Term Intermittent Hypoxia

**DOI:** 10.1371/journal.pone.0131068

**Published:** 2015-06-24

**Authors:** Christian Giordano, Christian Lemaire, Tong Li, R. John Kimoff, Basil J. Petrof

**Affiliations:** 1 Meakins-Christie Laboratories and Respiratory Division, McGill University, Montreal, Quebec, Canada; 2 Program for Translational Research in Respiratory Diseases, McGill University Health Centre Research Institute, Montreal, Quebec, Canada; University of Louisville School of Medicine, UNITED STATES

## Abstract

**Background:**

Short-term intermittent hypoxia (IH) is common in patients with acute respiratory disorders. Although prolonged exposure to hypoxia induces atrophy and increased fatigability of skeletal muscle, the response to short-term IH is less well known. We hypothesized that the diaphragm and limb muscles would adapt differently to short-term IH given that hypoxia stimulates ventilation and triggers a superimposed exercise stimulus in the diaphragm.

**Methods:**

We determined the structural, metabolic, and contractile properties of the mouse diaphragm after 4 days of IH (8 hours per day, 30 episodes per hour to a FiO2 nadir=6%), and compared responses in the diaphragm to a commonly studied reference limb muscle, the tibialis anterior. Outcome measures included muscle fiber size, assays of muscle proteolysis (calpain, ubiquitin-proteasome, and autophagy pathways), markers of oxidative stress and mitochondrial function, quantification of intramyocellular lipid and lipid metabolism genes, type I myosin heavy chain (MyHC) expression, and in vitro contractile properties.

**Results:**

After 4 days of IH, the diaphragm alone demonstrated significant atrophy (30% decrease of myofiber size) together with increased LC3B-II protein (2.4-fold) and mRNA markers of the autophagy pathway (LC3B, Gabarapl1, Bnip3), whereas active calpain and E3 ubiquitin ligases (MuRF1, atrogin-1) were unaffected in both muscles. Succinate dehydrogenase activity was significantly reduced by IH in both muscles. However, only the diaphragm exhibited increased intramyocellular lipid droplets (2.5-fold) after IH, along with upregulation of genes linked to activated lipid metabolism. In addition, although the diaphragm showed evidence for acute fatigue immediately following IH, it underwent an adaptive fiber type switch toward slow type I MyHC-expressing fibers, associated with greater intrinsic endurance of the muscle during repetitive stimulation in vitro.

**Conclusions:**

Short-term IH induces preferential atrophy in the mouse diaphragm together with increased autophagy and a rapid compensatory metabolic adaptation associated with enhanced fatigue resistance.

## Introduction

Intermittent hypoxia (IH) occurs in several respiratory disorders, including obstructive sleep apnea (OSA) and diseases such as emphysema and pulmonary fibrosis. As a general rule, chronic ongoing exposure to IH has been considered detrimental, as it has been linked to adverse cardiovascular, cognitive, and metabolic outcomes [1]. On the other hand, there has been considerable interest over whether more short-term IH might have benefits, particularly with respect to training effects in skeletal muscle [2]. In this regard, there is evidence that skeletal muscle hypertrophy, strength, and metabolic efficiency can be enhanced when IH is introduced into a training regimen, although this has not been uniformly reported and remains controversial [2]. In addition, while impaired inspiratory muscle function in OSA patients has been reported [3], other authors have failed to find evidence of diaphragmatic dysfunction in these patients [4].

Although most studies have focused on situations characterized by prolonged exposure to IH such as OSA, there has been relatively little study of the short-term effects of IH on diaphragmatic function. However, short-term exposure to IH is a common occurrence in patients hospitalized with acute respiratory disorders such as pneumonia or pulmonary edema, and is typically most severe during sleep. It is almost certainly the case that the balance between benefits and adverse consequences of IH depends upon the pattern of hypoxia exposure, as well as its duration and severity [1]. Furthermore, tissue responses to IH are very likely to be not only organ-specific but also muscle-specific. Therefore, in the present study we determined the effects of short-term IH (8 hours/day for 4 days) on structural, metabolic, and contractile properties of the mouse diaphragm, and compared responses in the diaphragm to a commonly studied reference limb muscle, the tibialis anterior (TA). Our study reveals that the diaphragm undergoes unique structural and metabolic changes after short-term IH, which result in a preferentially atrophied but more fatigue-resistant muscle.

## Methods

### IH protocol

Male mice (8 weeks old, C57BL/10ScSnJ, Jackson Laboratories) were randomly divided into 2 groups. The IH group was exposed to hypoxia (FiO2 nadir = 6%) at a frequency of 30 times/hour, 8 hours per day during the light phase of the circadian cycle, for 4 consecutive days. On the first day of the protocol, mice were pre-adapted to less severe IH conditions (FiO2 nadir = 10%) for 3 hours prior to instituting the above protocol. IH was achieved by nitrogen injection into sealed cages as previously described [5,6]. Mice from the control group (Ctl) were exposed to the same experimental conditions but nitrogen was replaced by air. Five animals were housed per cage in a controlled pathogen-free facility throughout the course of study. At the end of the experimental protocol, mice were anesthetized with sodium pentobarbital (60 mg/kg body weight, IP) and sacrificed by cervical dislocation. All animal procedures were approved by the McGill University Animal Care and Use Committee, in accordance with the guidelines issued by the Canadian Council on Animal Care. This study followed the Animal Research: Reporting of *In Vivo* Experiments (ARRIVE) guidelines for reporting animal research. The ARRIVE checklist is available in S1 Table.

### Real time PCR quantification of gene expression

Total RNA was extracted from tissues or cells using isol-RNA lysis reagent (5prime: #2302700) according to the manufacturer’s protocol. RNA was treated with DNase I amplification grade (Invitrogen: #18068–015) and then quantified by spectrophotometric optical density measurement (NanoDrop 1000, Thermo scientific). The purified RNA was reverse transcribed to cDNA with iScript Reverse Transcription Supermix (Bio-Rad: #170–8841). Quantitative RT-PCR was performed using 5 ng of cDNA mixed with 10 μl Maxima SYBR Green/ROX qPCR Master Mixes (Thermo scientific: #K0222) and 1μl of 10μM primer mixes. RT-PCR was carried out for 40 cycles using a StepOne Plus Thermocycler (Applied Biosystems). Primer sequences are provided in S2 Table and mouse hypoxanthine-guanine phosphoribosyltransferase (HPRT)1 was used as an internal control. The relative quantification of gene expression was analyzed by the 2^-ΔΔCq^ method, and the results are expressed as n-fold difference relative to control.

### Western blotting

Protein lysates from muscles were centrifuged at 15,000g in 4°C for 20 min and the resulting supernatants were assayed for protein quantity using the Bradford method. The dilutions of primary (Calpain, Sigma: #C5736; LC3B, Cell signaling: #3868) and secondary (Calpain, Promega: #W4021; LC3B, Promega: #W4011) antibodies were made as per the manufacturers’ instructions. Assessment of protein carbonylation levels in muscle was performed using the Oxyblot Protein Oxidation Detection kit (Millipore: #S7150). Briefly, muscle lysates were incubated with 2,4-dinitrophenylhydrazine (DNPH) to derivatize the carbonyl groups present in oxidized proteins into 2,4-dinitrophenylhydrazone (DNP-hydrazone). Then, the proteins were separated by polyacrylamide gel electrophoresis followed by Western blotting with antibodies raised against DNP-hydrazone. All steps of the protocol were performed according to manufacturer’s instructions. Acquisition and quantification of density for specific protein bands was performed by the ChemiDoc MP Imaging System (Bio-Rad). Ponceau S solution (Sigma: #P7170) was used to control for protein loading.

### Histological analysis

Excised muscles (diaphragm and TA) were quickly frozen in liquid nitrogen-cooled 2-methylbutane (Fisher, #O3551) and stored at –80°C. For general morphology, 8μm-thin cryostat sections were stained with haematoxylin and eosin (H&E) according to standard protocols. Images were photographed using an Olympus BX51 microscope with a QImaging Retiga 2000R camera system. A grid containing fixed dimension squares (352μm x 352μm) was randomly applied onto each photographic image. To quantify the cross-sectional area of individual myofibers, measurements were made on myofibers contained within 5 randomly chosen squares, using ImageJ software [7]. For each tissue section analyzed, approximately 500 muscle fibers were evaluated. Type I myofibers were detected by immunofluorescence on transverse muscle sections using an overnight incubation at 4°C with a primary antibody (Developmental Studies Hybridoma Bank: #BA-F8) raised against the mouse slow type I myosin heavy chain (MyHC) isoform. After 4 washes with PBS, muscle sections were incubated with a fluorescent anti-mouse IgG antibody (Molecular Probes: A-21140) for 1 hour at room temperature, washed 4 times in PBS and mounted using Immu-mount (Thermo: #9990402). Images were acquired with a QImaging Retiga 2000R camera on an Olympus BX51 fluorescent microscope and the morphometric analysis of type I MyHC-positive myofibers was performed using ImageJ. To assess the level of intramyocellular lipid accumulation, muscle sections were stained with Oil red O. Lipid droplets were quantified using a threshold-based analysis method with ImageJ as previously described [8]. For each tissue section analyzed, the integrated density of the staining was reported to the total section area and compared to the control value.

### Measurement of SDH activity

As previously described [9], frozen muscle samples were homogenized in 20 volumes of an ice-cold buffer (1mM EDTA, 50mM triethanolamine, pH 7.4). The homogenate was incubated on ice for 15 min, centrifuged (10 min, 14,000g, 4°C), and the supernatant was used for enzymatic activity measurements. Succinate dehydrogenase (SDH) activity was measured at 600 nm to detect the reduction of 2,6-Dichloroindophenol (DCIP) induced by the SDH-dependent oxidation of succinate and decylubiquinone. The assay was performed at 30°C in a KPi-EDTA buffer containing 1mg/ml BSA, 0.24mM KCN, 4μM rotenone, 0.4μM antimycine A, 100μM decylubiquinone, 100μM DCIP, 10mM succinate, and 0.2mM ATP at pH 7.4. SDH activity was calculated by the difference of DCIP reduction (μmol sec^-1^) with and without malonate, an inhibitor of SDH activity. Data were expressed per milligram of muscle tissue.

### Evaluation of diaphragm contractility

Diaphragm strips were dissected and placed into equilibrated (95% O_2_-5% CO_2_; pH 7.38) Krebs solution as previously described [9]. After attaching the muscle to a force transducer/length servomotor system (model 300B; dual mode; Cambridge Technology, Watertown, MA), optimal length (*Lo*) was determined. The force-frequency relationship was measured by sequential supramaximal stimulation for 1 sec at 10, 30, 50, 100 and 150 Hz, with 2 min between each stimulation train. Muscle force was normalized to cross-sectional area and expressed as Newtons/cm^2^. To assess fatigue resistance, muscles were intermittently stimulated (330 ms trains at 30 Hz each second) and the decrement in force production was continuously recorded over a period of 160 seconds.

### Statistical analysis

For each experiment, we evaluated the sample size according to an estimated standard deviation of samples based on pilot experiments and/or prior studies. Data are expressed as mean values ± SE and the significance of differences between the Ctl and IH groups was analyzed with a Student’s unpaired t-test, or ANOVA with post-hoc application of the Tukey test to adjust for multiple comparisons when appropriate. Statistical significance was set at P<0.05.

## Results

### Intermittent hypoxia promotes atrophy in the diaphragm but not limb muscle

**Fig 1:**
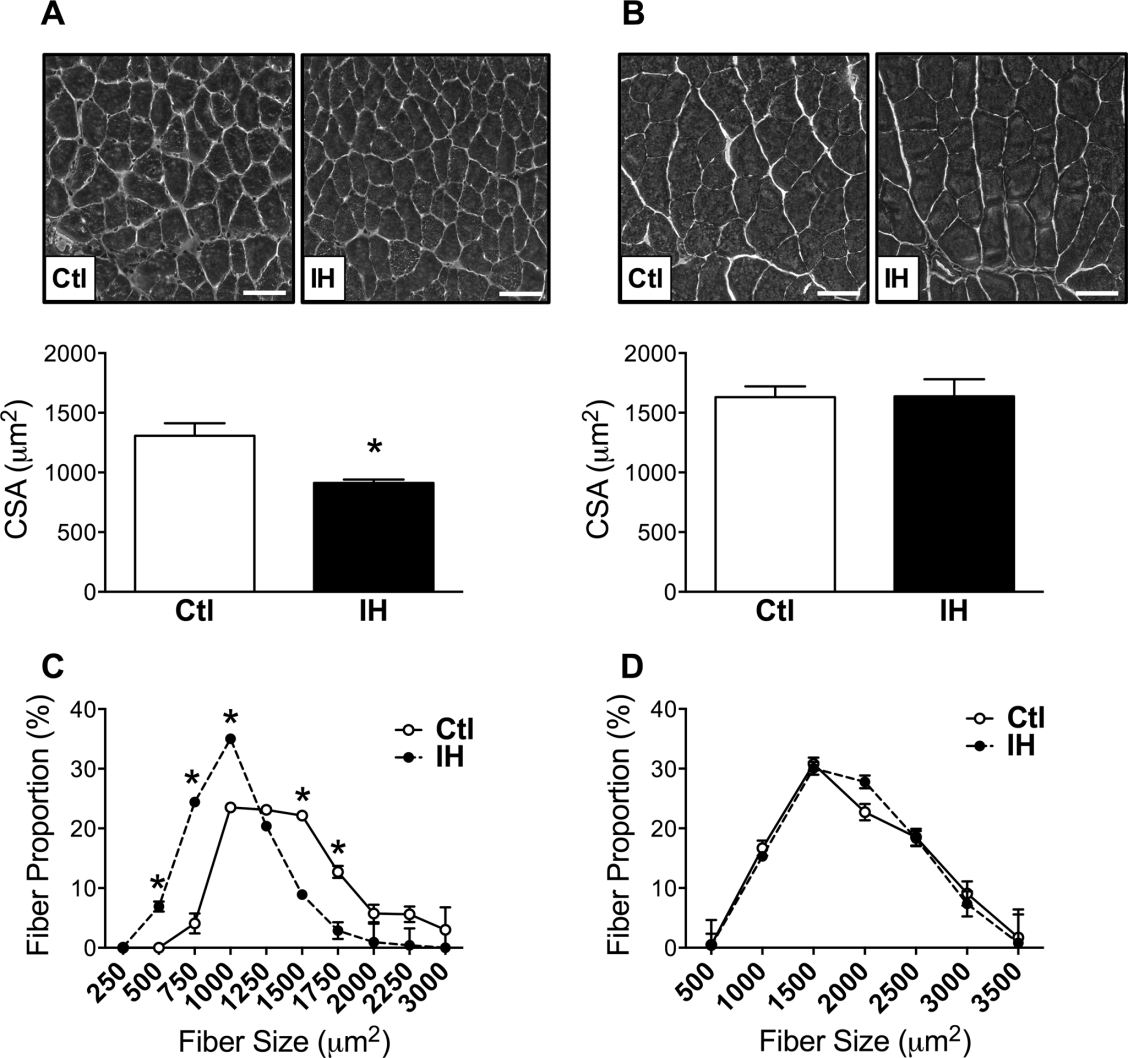
Myofiber atrophy in diaphragm but not in limb muscle after IH. Representative images and quantification of cross-sectional area (CSA) in: (A) diaphragm and (B) tibialis anterior limb muscle, of control (Ctl) and hypoxia-exposed (IH) mice. Frequency distributions of fiber size in: (C) diaphragm and (D) limb muscle. Data are expressed as means (± SE) of 5 mice per group. * p<0.01 compared to Ctl by t-test. Scale bar = 50μm.

After 4 days of IH exposure, the mean cross-sectional area of diaphragm myofibers was reduced by approximately 30% compared to the normoxic control (Ctl) group (Fig 1A), but no significant effect of IH on fiber size was observed in the TA limb muscle (Fig 1B). Consistent with this result, the frequency distribution of cross-sectional area values for individual diaphragm myofibers demonstrated a clear leftward shift toward an increased proportion of smaller fibers in the IH group (Fig 1C), whereas in the case of the limb muscle fiber size distribution curves did not differ between the Ctl and IH groups (Fig 1D). Hence, short-term exposure to IH led to preferential atrophy of the diaphragm.

### Differential activation of autophagy in the diaphragm and limb muscle

**Fig 2:**
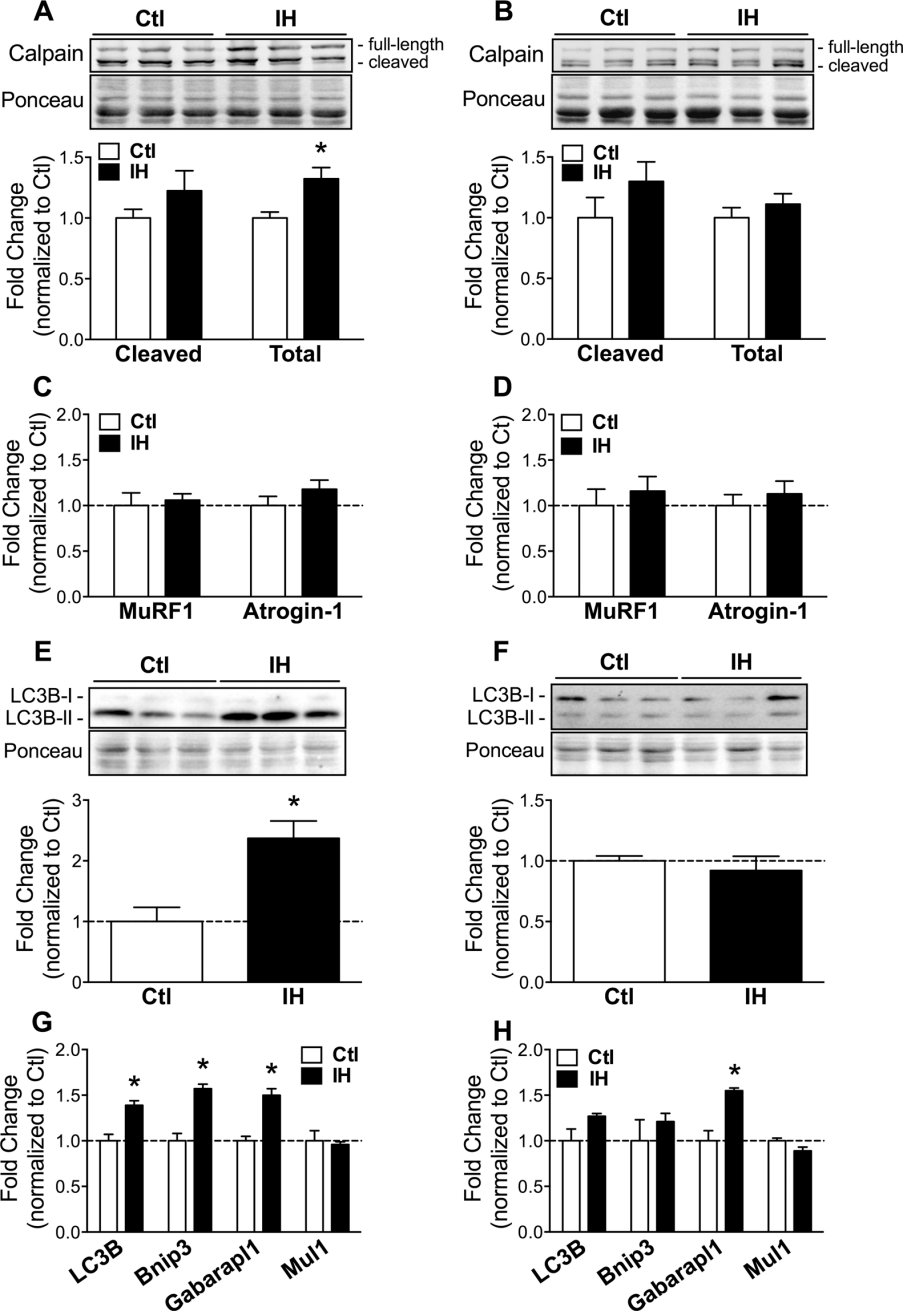
Influence of IH on proteolysis pathways in diaphragm and limb muscle. Western blot quantification of cleaved and total calpain proteins in: (A) diaphragm and (B) limb muscle, of Ctl and IH mice. Real-time PCR quantification of E3 ubiquitin ligases MuRF1 and atrogin-1 mRNA in: (C) diaphragm and (D) limb muscle. Western blot analysis and quantification of LC3B-II protein in: (E) diaphragm and (F) limb muscle. Real-time PCR quantification of autophagy-related gene expression in: (G) diaphragm and (H) limb muscle. Data are expressed as means (± SE) of 3–5 mice per group. * p<0.01 compared to Ctl by t-test. Abbreviations used: MuRF = Muscle ring finger; LC3B = Microtubule-associated protein 1 light chain 3; Bnip = Bcl2/adenovirus E1B 19 kDa interacting protein; Gabarapl = GABA(A) receptor-associated protein like; Mul = Mitochondrial ubiquitin ligase.

To identify the potential molecular mechanisms underlying preferential atrophy of the diaphragm during IH, we investigated the calpain [10], ubiquitin-proteasome [11], and autophagy [12] pathways of muscle proteolysis. Western blot analysis revealed a significant increase of total calpain protein in diaphragm (Fig 2A) but not TA limb muscle (Fig 2B) exposed to IH. However, neither the diaphragm nor the limb muscle showed a significant increase in the cleaved (active) form of calpain, arguing against a major role for this pathway in the preferential diaphragmatic atrophy induced by IH. Along these same lines, real-time quantitative PCR did not demonstrate significant changes in expression levels of the muscle-specific E3 ubiquitin ligases, muscle ring finger 1 (MuRF1) and atrogin-1, in either the diaphragm (Fig 2C) or the limb muscle (Fig 2D) following IH. On the other hand, levels of microtubule-associated protein 1 light chain 3B-II (LC3B-II) protein, a biochemical marker of autophagosomes, were increased approximately 2.4-fold in the diaphragms (Fig 2E) of IH-exposed mice while no significant change was discernable in the limb muscle (Fig 2F). Furthermore, quantitative PCR for several prototypical genes involved in autophagy revealed a significant increase of LC3B, Bcl2/adenovirus E1B 19 kDa interacting protein 3 (Bnip3) and GABA(A) receptor-associated protein like 1 (Gabarapl1) in the diaphragm (Fig 2G), whereas only Gabarapl1 expression was increased in limb muscle (Fig 2H). Taken together, these findings implicate autophagy as being a major proteolysis pathway involved in preferential atrophy of the diaphragm during IH.

### Similar oxidative stress responses in both muscles

**Fig 3:**
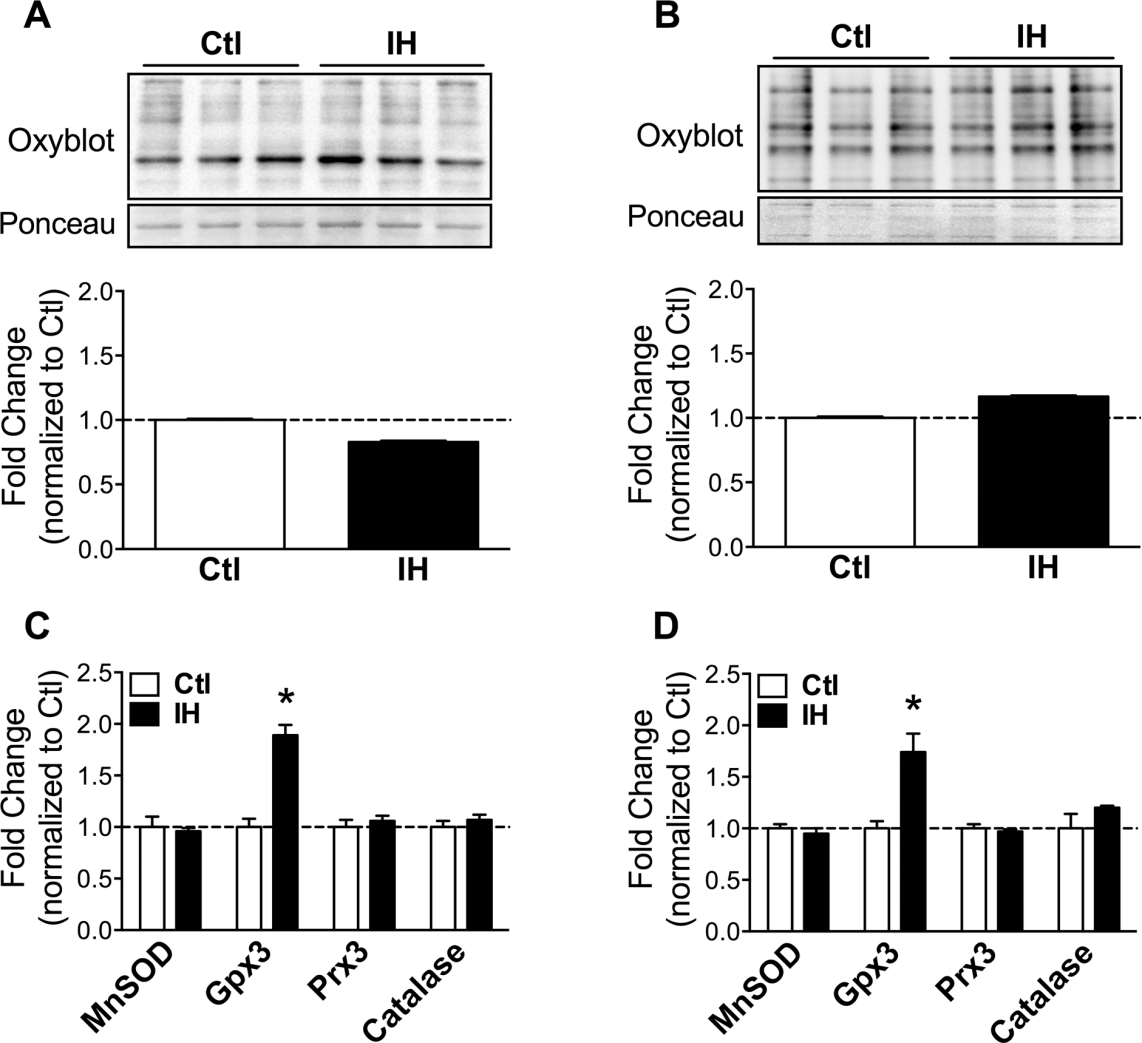
Oxidative stress markers in diaphragm and limb muscle after IH. Western blot quantification of carbonylated proteins in: (A) diaphragm and (B) limb muscle, of Ctl and IH mice. Real-time PCR quantification of anti-oxidant gene expression in: (C) diaphragm and (D) limb muscle. Data are expressed as means (± SE) of 4–5 mice per group. * p<0.01 compared to Ctl by t-test. Abbreviations used: MnSOD = Manganese superoxide dismutase; Gpx = Glutathione peroxidase; Prx = Peroxiredoxin.

Oxidative stress has been reported as a frequent response to hypoxia [13–15], and has additionally been linked to activation of muscle proteolysis pathways including autophagy [16,17]. To ascertain whether IH led to increased oxidative stress in diaphragm and limb muscle, we quantified oxidative protein modifications (total carbonylated proteins) by Western blotting. No significant increase of carbonylated proteins after IH was detected in either the diaphragm (Fig 3A) or limb muscle (Fig 3B). To further characterize oxidative stress, we assessed by real-time PCR the expression of anti-oxidant genes including manganese superoxide dismutase (MnSOD), glutathione peroxidase 3 (Gpx3), peroxiredoxin 3 (Prx3), and catalase. None of these genes demonstrated significant changes in expression with the exception of Gpx3, which was mildly upregulated in both diaphragm (Fig 3C) and limb muscle (Fig 3D). These data suggest that oxidative stress was mild and/or well-compensated by anti-oxidant defense mechanisms in both muscles.

### Metabolic remodeling with reduced SDH activity in both muscles

**Fig 4:**
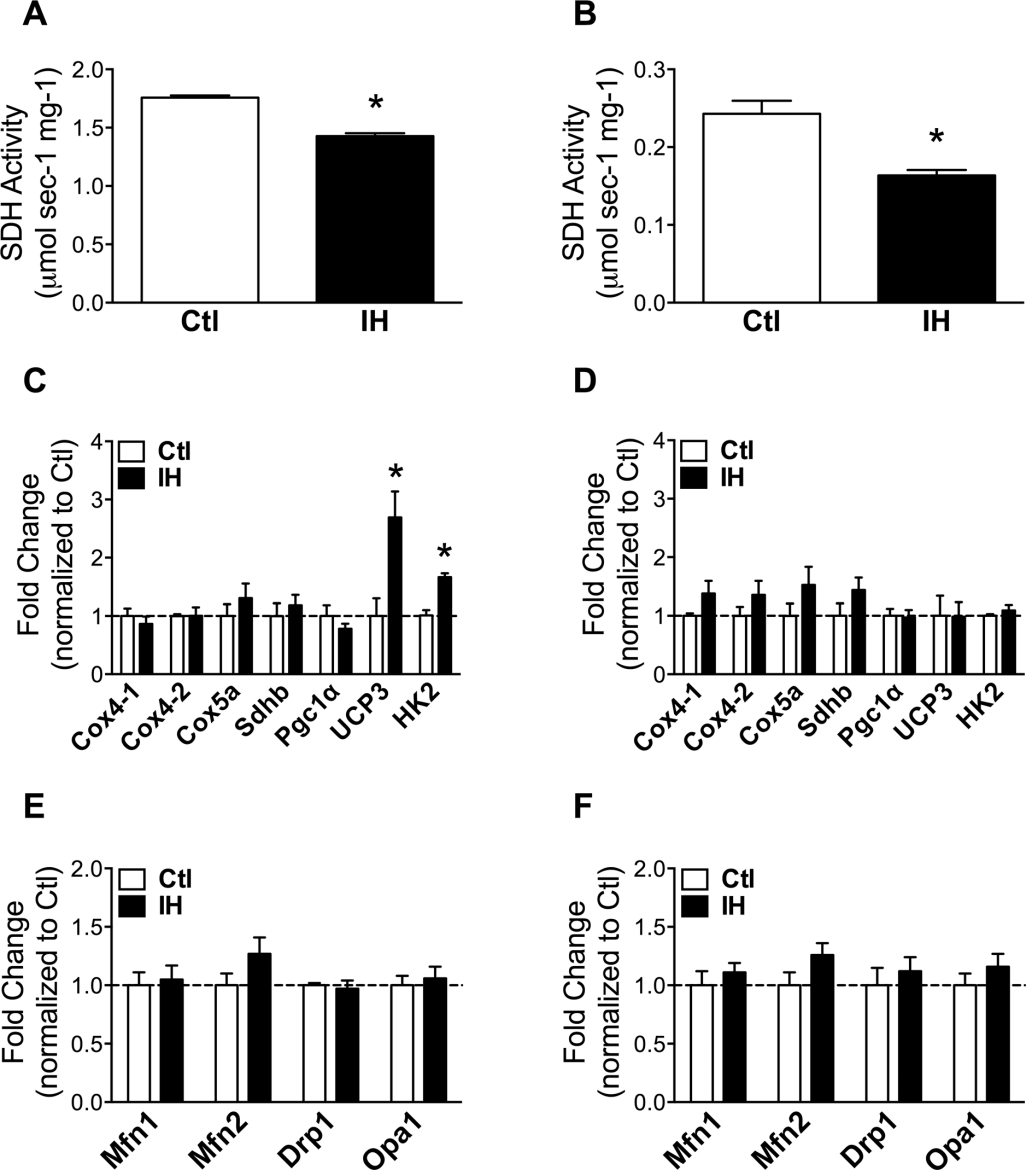
Indices of mitochondrial function in diaphragm and limb muscle after IH. Succinate dehydrogenase (SDH) activity in: (A) diaphragm and (B) limb muscle, of Ctl and IH mice. Gene expression levels of oxidative and glycolytic markers in: (C) diaphragm and (D) limb muscle. Quantification of mitochondrial dynamics gene expression in: (E) diaphragm and (F) limb muscle. Data are expressed as means (± SE) of 4–5 mice per group. * p<0.01 compared to Ctl by t-test. Abbreviations used: Mfn = Mitofusin; Drp = Dynamin-related protein; Opa = Optic atrophy; Cox = Cytochrome C oxidase; Sdh = Succinate dehydrogenase; Pgc = PPARgamma coactivator; UCP = Uncoupling protein; HK = Hexokinase.

Succinate dehydrogenase (SDH) activity and gene expression levels of electron transport chain components (cytochrome C oxidases: Cox4-1, Cox4-2, Cox5a), the SDH subunit B (Sdhb), as well as the key regulator of mitochondrial biogenesis PPARγ coactivator 1α (Pgc1α), were evaluated as indices of mitochondrial oxidative capacity. As expected given its more oxidative profile, basal SDH activity in the diaphragm was approximately 7-fold (p = 9.4E-08) higher than in the TA limb muscle. Following exposure to IH, SDH activity was significantly reduced in both the diaphragm (Fig 4A) and the limb muscle (Fig 4B). In addition, while mRNA expression levels remained unchanged for electron transport chain components and Pgc1α in both muscles after IH, the diaphragm alone demonstrated a significant increase of uncoupling protein 3 (UCP3), previously implicated in fatty acid oxidation [18], as well as the glycolytic enzyme hexokinase-2 (HK2) (Fig 4C and 4D). Genes involved in mitochondrial structural remodeling via fusion and fission such as mitofusin (Mfn) 1 and 2, optic atrophy 1 (Opa1), and dynamin-related protein 1 (Drp1) [19], were unaffected in their expression levels by IH in both muscles (Fig 4E and 4F).

### Preferential lipid droplet accumulation and activation of lipid metabolism in the diaphragm

**Fig 5:**
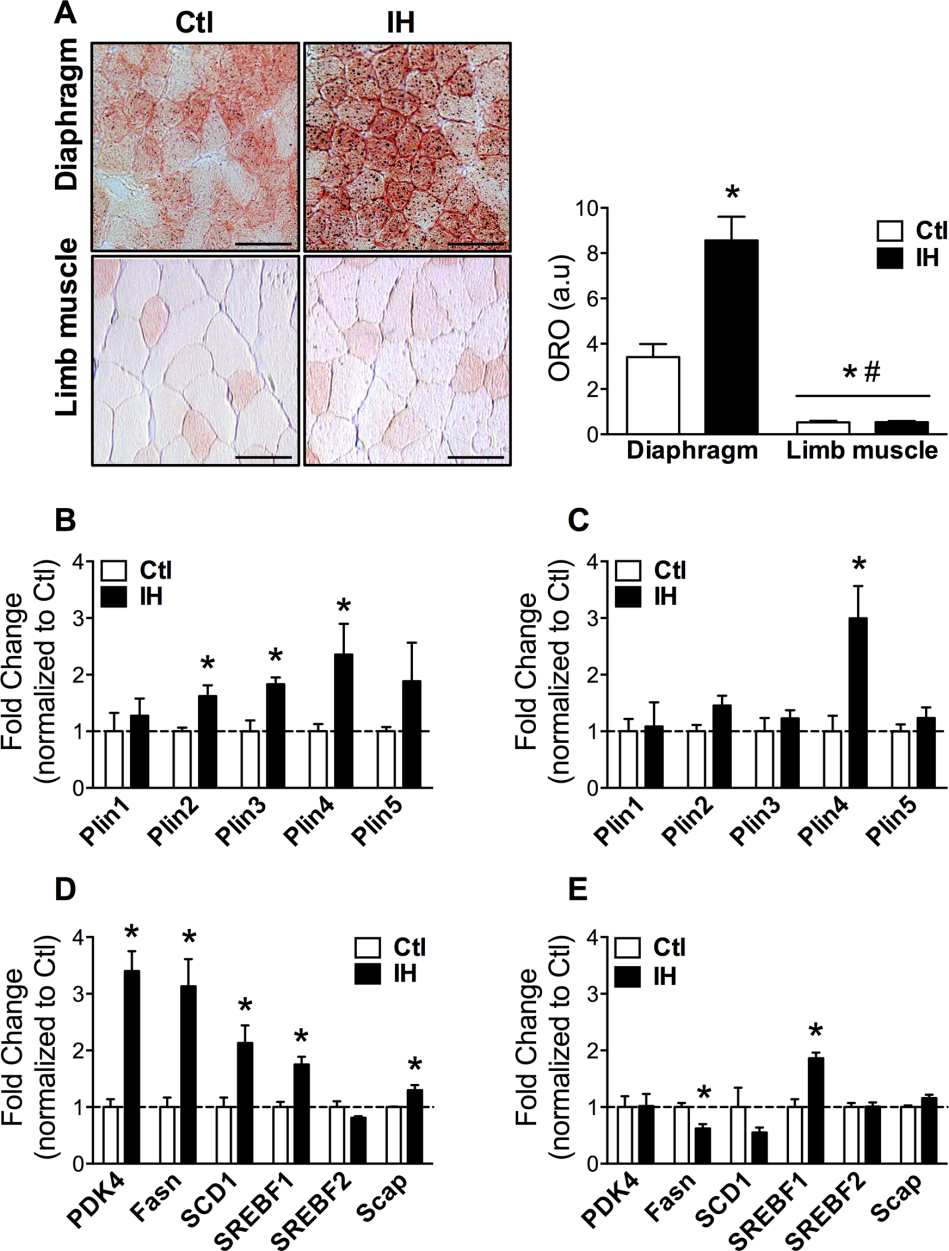
IH promotes lipid droplets and activation of lipid metabolism genes in the diaphragm. Representative Oil-red O images (left panel) and quantification of lipid droplet staining (right panel) in the diaphragm and limb muscle in (A). Quantification of expression levels for Plin isoforms (B and C) and other lipid metabolism pathway genes (D and E) in: (B and D) diaphragm and (C and E) limb muscle. Data are expressed as means (± SE) of 4–5 mice per group. For (A), * p<0.01 compared to Ctl diaphragm and # p<0.01 compared to IH diaphragm by ANOVA; for (B-E), * p<0.01 compared to Ctl by t-test. Scale bar = 100μm. Abbreviations used: Plin = Perilipin; PDK = Pyruvate dehydrogenase kinase; Fasn = Fatty acid synthase; SCD = Stearoyl-coenzyme A desaturase; SREBF = Sterol regulatory element binding transcription factor; Scap = SREBF cleavage-activating protein.

Metabolic remodeling was further assessed by quantifying intramyocellular lipid droplet content. Consistent with its more oxidative profile, basal lipid droplet levels in the diaphragm were approximately 6-fold (p = 0.001) higher than in the limb muscle (Fig 5A). Following IH, the diaphragm showed an approximate 2.5-fold increase of lipid droplets, whereas no significant changes were found in the limb muscle (Fig 5A). Members of the perilipin (Plin) family of lipid droplet-associated proteins, previously implicated in lipid processing [20], were also differentially regulated in the two muscles. Hence several Plin genes (Plin 2, 3, and 4) were significantly upregulated in the diaphragm after IH (Fig 5B), while only Plin4 was modulated by IH in the limb muscle (Fig 5C). Furthermore, expression levels of other lipid metabolism pathway genes such as pyruvate dehydrogenase kinase 4 (PDK4), fatty acid synthase (Fasn), stearoyl-coenzyme A desaturase 1 (SCD1), sterol-regulatory element binding transcription factor 1 (SREBF1), and SREBF cleavage-activating protein (Scap) were all increased in the diaphragm after IH (Fig 5D). In contrast, Fasn was significantly downregulated and only SREBF1 was increased by IH in the limb muscle (Fig 5E). These findings collectively suggest that IH exposure is associated with a preferential activation of lipid metabolism in the diaphragm following short-term IH.

### Increased type I myosin heavy chain (MyHC) and fatigue resistance in the diaphragm

**Fig 6:**
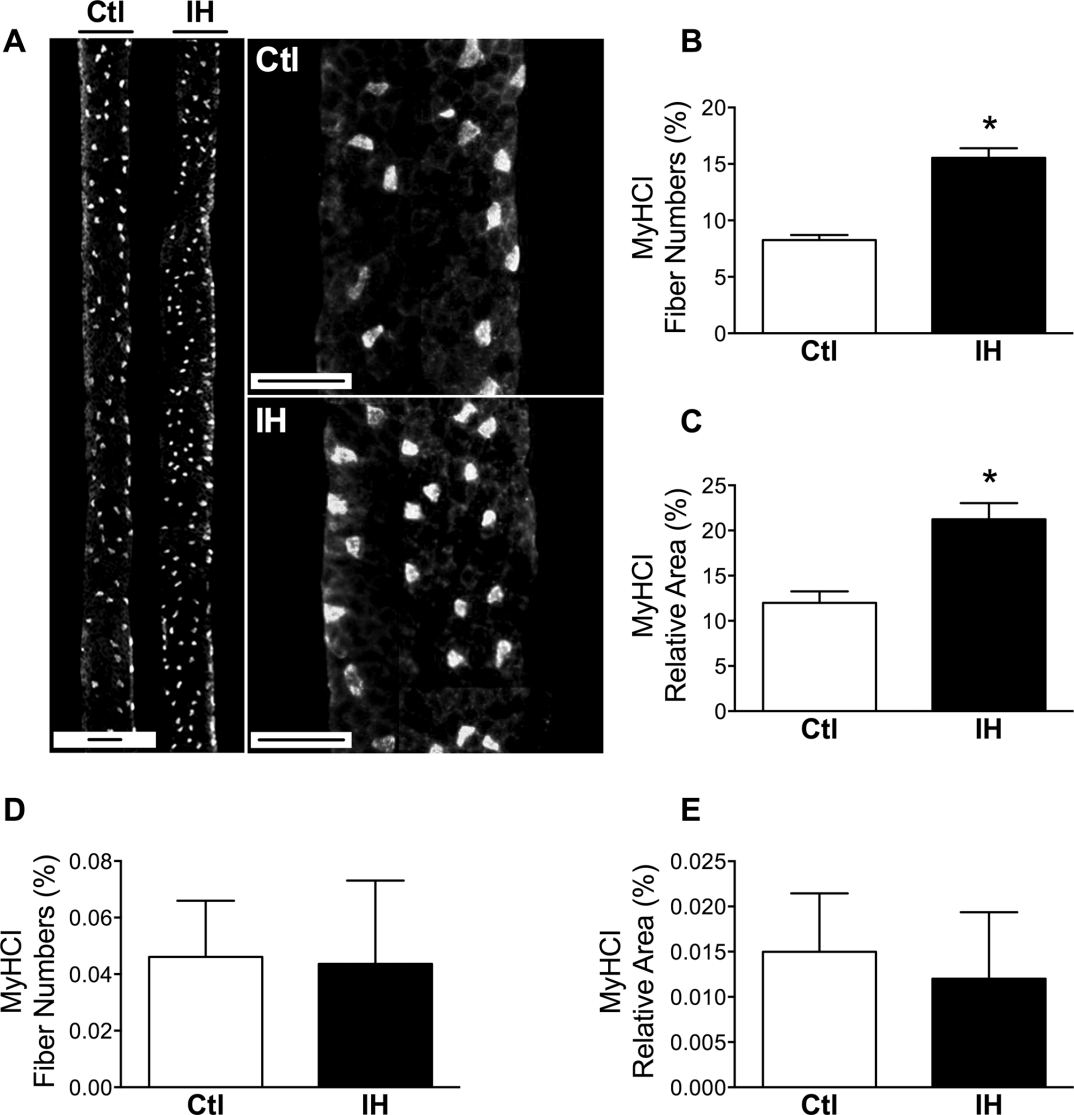
Type I myofibers are increased by IH in diaphragm but not limb muscle. (A) Low and high magnification images of Ctl and IH diaphragm sections immunostained for type I myosin heavy chain (MyHCI). (B) Proportion of myofibers expressing type I MyHC in the diaphragm. (C) Quantification of the relative area contribution of type I MyHC-expressing fibers to total tissue cross-sectional area of the diaphragm. (D) Proportion of MyHCI-positive myofibers in the limb muscle. (E) Quantification of the relative area contribution of MyHCI-positive fibers to total tissue cross-sectional area of the limb muscle. Data are expressed as means (± SE) of 5 mice per group. * p<0.01 compared to Ctl by t-test. Scale bar = 100μm.

We next wished to determine whether the metabolic remodeling responses observed in the diaphragm following IH might be associated with a fiber type switch, since type I fibers typically have high SDH activity and also contain higher amounts of lipid [21]. Using type I MyHC immunostaining (Fig 6A), we observed a significant increase in the percentage of type I myofiber numbers (Fig 6B) as well as their relative contribution to total diaphragm surface area (Fig 6C) in the group subjected to IH. In contrast, for the limb muscle no significant changes were found after IH in either type I myofiber number percentage (Fig 6D) or the contribution of these fibers to total muscle cross-sectional area (Fig 6E).

**Fig 7:**
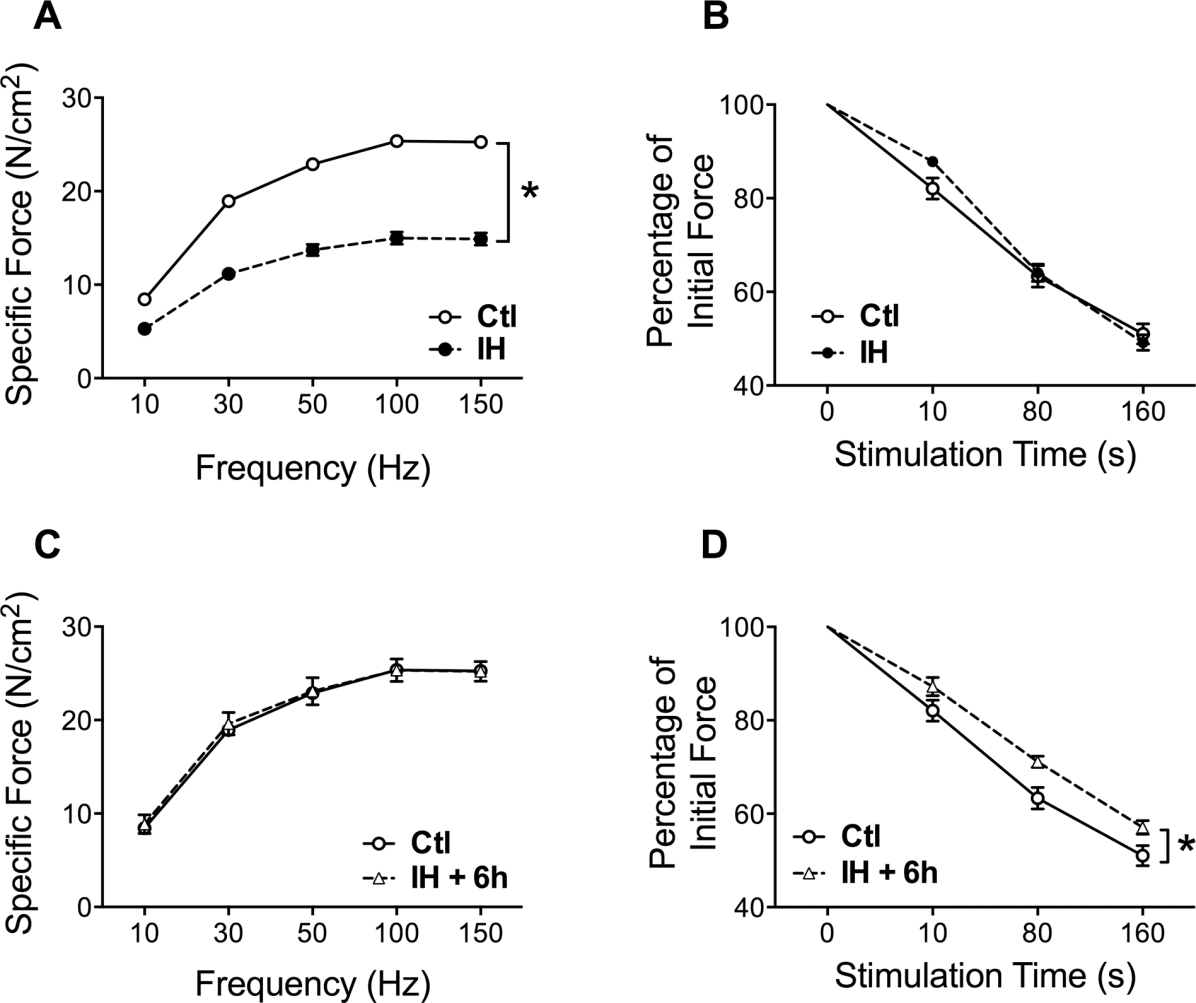
IH induces acute fatigue and greater intrinsic endurance capacity of the diaphragm. (A) Force-frequency curves and (B) force decrement during repetitive contractions to induce fatigue, in Ctl and IH diaphragms immediately after the last hypoxia session on day 4. (C) Force-frequency curves and (D) force decrement during repetitive contractions of the diaphragm 6 hours after the last hypoxia session on day 4. Data are expressed as means (± SE) of 5–10 mice per group. * p<0.01 compared to Ctl by ANOVA.

Given that MyHC fiber type composition is a major determinant of fatigue resistance, we additionally ascertained whether the increased contribution of slow-twitch type I myofibers impacted upon contractile properties of the muscle. This was done immediately following termination of the last IH session, as well as 6 hours later to allow recovery from any fatigue which may have been induced by the IH exposure. Immediately post-IH, the maximal force-generating capacity of the diaphragm was reduced by approximately 40% as compared to Ctl mice (Fig 7A), whereas no significant differences in the rate of diaphragmatic force loss during repetitive stimulation (i.e., fatigue resistance) were detected between the two groups at this time (Fig 7B). In contrast, when the diaphragm was examined 6 hours later, maximal force production in the IH group was completely restored to normal Ctl values (Fig 7C), implying that the diaphragm had recovered from a state of muscle fatigue induced by the IH protocol. Under these conditions, the now “rested” diaphragm of the IH group demonstrated greater maintenance of force during repeated stimulation than the Ctl group (Fig 7D), indicating greater intrinsic resistance to fatigue and thus consistent with the increased proportion of type I fibers found in the muscle.

## Discussion

In the present study, we investigated whether short-term IH (8 hours/day for 4 days) in mice differentially alters diaphragm and limb muscle (TA) properties with regard to myofiber structure and function. We also determined the relationship of these changes to canonical pathways linked to proteolysis, oxidative stress, and mitochondrial metabolism. The main findings of our study are: 1) IH induced preferential atrophy of the diaphragm, which correlated with greater activation of the autophagy but not calpain or muscle-specific E3 ubiquitin ligase pathways; 2) a similar anti-oxidant response and decreased SDH activity were found in both muscles; and 3) the diaphragm demonstrated a unique metabolic profile characterized by greater lipid droplet accumulation and activation of lipid metabolism pathway genes, an increase in type I MyHC-expressing fibers, and enhanced fatigue resistance despite reduced SDH activity of the muscle.

It is likely that the similar responses to IH observed in the two muscles, such as the increase of anti-oxidant Gpx3 expression and the reduction of SDH activity found in both diaphragm and TA, are primarily related to hypoxia per se. Oxidative stress is a well-described consequence of hypoxia in various organ systems and cell types [14,15]. In our study, this appears to have been relatively well-compensated by anti-oxidant mechanisms insofar as there was no measurable excess of protein carbonylation in the muscles after IH, although we cannot exclude a mild degree of oxidative stress. Shortt et al [13] recently reported similar findings in diaphragms of rats exposed to 2 weeks of IH, in which the GSSG/GSH ratio was increased but without evidence for increased lipid peroxidation. In addition, the decreased SDH activity which we observed in the two muscles is consistent with the downregulation of mitochondrial content and/or activity previously reported in mouse hindlimb muscles exposed to continuous hypoxia (FiO2 = 0.10) over 4 weeks [22] as well as similar findings in other studies involving sustained rather than intermittent hypoxic exposure [23].

On the other hand, a number of divergent responses were observed between the diaphragm and limb muscle, which could result from their different activity levels during the hypoxic stimulus (i.e., greater in the diaphragm), or alternatively be due to muscle-specific differences in their intrinsic properties (fiber type, etc.). For example, whereas no atrophy occurred in the limb muscle, the diaphragm underwent an approximate 30% reduction in muscle fiber size during the IH protocol. To decipher the molecular mechanisms involved in this atrophy, we investigated 3 major proteolytic systems: the calcium-activated calpain, ubiquitin-proteasome and autophagy pathways. Our data suggest that amongst these proteolytic systems, the autophagy-lysosomal pathway is most likely to account for the observed differential atrophy responses between diaphragm and limb muscle. Indeed, it is well established that hypoxia can trigger cellular autophagy in a hypoxia-inducible factor (HIF)-dependent manner [16]. Interestingly, autophagy has been reported to be protective against cardiac dysfunction induced by IH [17], and may have played a beneficial role in the metabolic remodeling observed in our study by eliminating dysfunctional mitochondria (mitophagy) [12].

Exercise has also been shown to increase basal autophagy, which appears necessary for optimal muscle adaptation and improvement of physical performance [24]. We posit that the increased work of breathing during IH is equivalent to an exercise stimulus for the diaphragm, which when combined with hypoxia-related signaling serves to reinforce cellular activation of autophagy and thereby induce atrophy. Alternatively, greater atrophy in the diaphragm could be due to a greater intrinsic sensitivity of its fibers to hypoxia-induced muscle atrophy. The diaphragm and TA muscles are both composed predominantly of type II fast-twitch fibers in the mouse (approximately 90% and 100%, respectively), but the diaphragm fibers are inherently more oxidative and fatigue resistant [25]. However, in a recent comparison of oxidative (soleus) and glycolytic (extensor digitorum longus, EDL) limb muscle responses to continuous hypoxia (FiO2 = 0.08 for 4 or 21 days), muscle atrophy and autophagy activation were significantly more pronounced in the EDL [26]. Therefore, it appears unlikely that intrinsic fiber type differences account for preferential atrophy of the diaphragm in our study, since its more oxidative fibers should in principle confer greater protection against hypoxia-induced atrophy.

This study provides novel evidence for altered lipid metabolism in the diaphragm after IH. The diaphragm demonstrated a 2.5-fold increase of lipid droplet accumulation as compared to normoxic controls, whereas the limb muscle did not show any significant alteration. Consistent with this observation, we documented an upregulation of key genes involved in fatty acid biosynthesis such as Fasn and its transcriptional regulator SREBP1, SCD1, and the SREBP-cleavage activating protein Scap in the diaphragm. In contrast, most of these lipogenic markers were either downregulated (Fasn) or remained unchanged (SCD1, Scap) in limb muscle. In addition, Plin2 and Plin3 were preferentially induced by IH in the diaphragm, suggesting active processing of intramyocellular lipids since the lipid droplet-associated Plin proteins appear to be markers of lipid utilization during exercise [27,28]. Furthermore, the specific induction of both PDK4 and UCP3 in the diaphragm is similarly consistent with a metabolic shift towards greater fatty acid oxidation [18,29]. Therefore, our data collectively support increased lipogenesis, fatty acid storage and utilization in the diaphragm after IH, which mimics aspects of the metabolic signature reported in athletes’ muscles [30,28,31].

The nature and functional consequences of intramyocellular lipid accumulation are poorly understood, being linked to insulin resistance in obesity [32] but considered as a potentially beneficial adaptation in the muscles of endurance-trained athletes [33]. Oxidative muscle fibers store greater amounts of lipids to support their more sustained energy demands [21], which explains the higher basal levels of lipid droplets observed in the diaphragm as compared to the limb muscle in normoxic animals. Lipid droplets have long been recognized to accumulate in hypoxic cells [34]. Although this could be due to a reduced ability to oxidize fatty acids, there is evidence that this response is not solely a consequence of unused substrate accumulation, but is actively promoted in many cell types including neurons [35], liver [36], heart [37], immune cells [38] and adipose tissue [39–41]. This may be at least partly related to the phenomenon of aerobic glycolysis (also known as the Warburg effect), which can occur when mitochondrial pyruvate metabolism is inhibited by hypoxia, exposure to reactive oxygen species (ROS) or other factors [42], leading to its alternative conversion into lactate. The induction of HK2 mRNA in the diaphragm after IH is consistent with this hypothesis given its role as an important mediator of the Warburg effect [43]. Increased expression of PDK4 provides further supportive evidence, since PDK4-dependent phosphorylation of the pyruvate dehydrogenase enzyme complex inhibits pyruvate conversion into acetyl-CoA in mitochondria [29]. Under such conditions, glutamate-dependent anapleurosis can also promote reductive metabolism of α-ketoglutarate to supply citrate for lipid biosynthesis [44]. Although the precise biochemical nature of the lipids which accumulate in hypoxic cells are not well delineated, these lipids may serve other functions beyond simply providing an alternative substrate for ATP production.

A shift toward greater lipid utilization and type I MyHC expression in the diaphragm following IH may initially seem counterintuitive given the simultaneous reduction of mitochondrial complex II (i.e., SDH) activity. However, a recent study reported that myotubes exposed to short-term hypoxia in vitro similarly demonstrated upregulated type I MyHC expression despite a concomitant reduction in oxidative phosphorylation complex proteins [45]. In addition, the increased diaphragm muscle activity triggered by IH could facilitate its ability to employ lipids as a fuel source through other mechanisms. In this regard, it has been reported that endurance training prevents hypoxia-induced decrements in mitochondrial fatty oxidation, possibly due to greater muscle carnitine palmitoyl transferase (mCPT)-1 activity and improved mitochondrial efficiency [46]. Previous studies have noted that mitochondrial content, respiratory capacity, and muscle endurance are not necessarily well correlated with one another [22,47]. Other investigators have found that SDH activity correlates less closely with fatigue resistance than does MyHC phenotype [48,49]. Improved diaphragmatic endurance without increased SDH activity was reported in the rat diaphragm after 6 weeks of chronic hypoxia exposure [50], and normal human volunteers trained under conditions of normobaric hypoxia demonstrated improved bicycle exercise endurance despite reduced quadriceps SDH activity, possibly mediated by a switch in COX isoforms [51] leading to enhanced oxygen affinity [52].

An adaptive response of the diaphragm leading to increased energetic efficiency after IH is supported by the combination of increased type I MyHC content and improved endurance properties of the muscle demonstrated in our study. Although diaphragmatic force-generating capacity was significantly depressed immediately after the last hypoxic session, it had completely recovered to normal 6 hours later. These findings are consistent with induction of diaphragmatic fatigue during the hypoxic sessions, which is not unexpected given the combination of reduced oxygen availability and increased work of breathing [53]. However, a key observation is that once the mice were returned to normoxia, the “rested” IH diaphragm muscle exhibited an improved capacity to resist fatigue during repetitive electrical stimulation in vitro. These physiological properties of the IH-exposed diaphragm are in keeping with the observed increase in fibers expressing type I MyHC, which are known to be more energetically efficient [54]. Furthermore, in view of the potential contribution of respiratory complex II to mitochondrial ROS production [55], the decrease in SDH activity could conceivably play a role in limiting muscle fatigue associated with ROS generation. However, a limitation of our investigation is that species differences may exist in these responses, since studies in rats have not found the same shift toward slow oxidative fibers in the diaphragm after IH [56], although a better maintenance of force production in IH-exposed diaphragms was reported [57].

In summary, our study shows that 4 days of IH induces major atrophy in the mouse diaphragm associated with increased autophagy, together with a compensatory metabolic adaptation linked to enhanced muscle endurance. These findings may have implications for acute pulmonary diseases associated with episodic hypoxemia. In addition, it is conceivable that short-term IH could be used as an adjunct to respiratory muscle training as a means of increasing diaphragmatic endurance provided that muscle atrophy can be avoided, and this possibility deserves further investigation.

## Supporting Information

S1 Table Animal Research: Reporting of *In Vivo* Experiments (ARRIVE) checklist of the study. (PDF)

S2 Table Sequences of PCR primers used for gene expression analysis. (DOC)
