# Supplementary material for: Autophagy-Associated Atrophy and Metabolic Remodeling of the Mouse Diaphragm after Short-Term Intermittent Hypoxia
Source: PLoS One. 2015 Jun 24;10(6):e0131068. doi: 10.1371/journal.pone.0131068 (PMC4480857; doi:10.1371/journal.pone.0131068)
Supplement: S2 Table — (DOC) [file pone.0131068.s002.doc]

| **Gene** | **Forward (5’ to 3’)** | **Reverse (5’ to 3’)** |  |
| --- | --- | --- | --- |
| **MuRF1** | ACCTGCTGGTGGAAAACA | AGGAGCAAGTAGGCACCTCA |  |
| **Atrogin-1** | GAGTGGCATCGCCCAAAAGA | TCTGGAGAAGTTCCCGTATAAGT |  |
| **LC3B** | CGATACAAGGGGGAGAAGCA | ACTTCGGAGATGGGAGTGGA |  |
| **Bnip3** | TGGGAGCAGCGTTCCAGCCT | TGTCTGGGAGCGAGGTGGGC |  |
| **Gabarapl1** | CATCGTGAGAAGGCTCCTA | ATACAGCTGGCCCATGGTAG |  |
| **Mul1** | CTGGGCACCAGTTCGATGG | GACAGCATAAGGCACACACTT |  |
| **MnSOD** | CAGACCTGCCTTACGACTATGG | CTCGGTGGCGTTGAGATTGTT |  |
| **Gpx3** | CCTTTTAAGCAGTATGCAGGCA | CAAGCCAAATGGCCCAAGTT |  |
| **Prx3** | GGTTGCTCGTCATGCAAGTG | CCACAGTATGTCTGTCAAACAGG |  |
| **Catalase** | AGCGACCAGATGAAGCAGTG | TCCGCTCTCTGTCAAAGTGTG |  |
| **Mfn1** | ACGTCGAGAACCTCCATGGGCA | CACGGGTCGTCCACGTCAGC |  |
| **Mfn2** | AGCACACAGTCCGGGCCAAG | GTCTTGCCGCTCTTCCCGCA |  |
| **Drp1** | CAGGAATTGTTACGGTTCCCTAA | CCTGAATTAACTTGTCCCGTGA |  |
| **Opa1** | TGGAAAATGGTTCGAGAGTCAG | CATTCCGTCTCTAGGTTAAAGCG |  |
| **Cox4-1** | AGTGTTGTGAAGAGTGAAGAC | GCGGTACAACTGAACTTTCTC |  |
| **Cox4-2** | GTTGACTGCTACGCCCAGCGC | CCGGTACAAGGCCACCTTCTC |  |
| **Cox5a** | TGCGAGCATGTAGACCGTTAAAT | GAGGTCCTGCTTTGTCCTTAACA |  |
| **Sdhb** | AATTTGCCATTTACCGATGGGA | AGTGTTGTGAAGAGTGAAGAC |  |
| **Pgc1α** | CAACAATGAGCCTGCGAACA | CTTCATCCACGGGGAGACTG |  |
| **UCP3** | CTGCACCGCCAGATGAGTTT | ATCATGGCTTGAAATCGGACC |  |
| **HK2** | GGGCATGAAGGGCGTGTCCC | TCTTCACCCTCGCAGCCGGA |  |
| **PDK4** | AGGGAGGTCGAGCTGTTCTC | GGAGTGTTCACTAAGCGGTCA |  |
| **Fasn** | GGAGGTGGTGATAGCCGGTAT | TGGGTAATCCATAGAGCCCAG |  |
| **SCD1** | TTCTTGCGATACACTCTGGTGC | CGGGATTGAATGTTCTTGTCGT |  |
| **SREBF1** | GCAGCCACCATCTAGCCTG | CAGCAGTGAGTCTGCCTTGAT |  |
| **SREBF2** | GCAGCAACGGGACCATTCT | CCCCATGACTAAGTCCTTCAACT |  |
| **Scap** | TGGAGCTTTTGAGACTCAGGA | TCGATTAAGCAGGTGAGGTCG |  |
| **Plin1** | GGGACCTGTGAGTGCTTCC | GTATTGAAGAGCCGGGATCTTTT |  |
| **Plin2** | GACCTTGTGTCCTCCGCTTAT | CAACCGCAATTTGTGGCTC |  |
| **Plin3** | ATGTCTAGCAATGGTACAGATGC | CGTGGAACTGATAAGAGGCAGG |  |
| **Plin4** | GTGTCCACCAACTCACAGATG | GGACCATTCCTTTTGCAGCAT |  |
| **Plin5** | TGTCCAGTGCTTACAACTCGG | CAGGGCACAGGTAGTCACAC |  |
| **HPRT1** | CGCAGTCCCAGCGTCGTGAT | CGAGCAAGTCTTTCAGTCCTGTCCA |  |

*List of Abbreviations*: MuRF=Muscle ring finger; LC3B=Microtubule-associated protein 1 light chain 3; Bnip= Bcl2/adenovirus E1B 19 kDa interacting protein; Gabarapl=GABA(A) receptor-associated protein like; Mul=Mitochondrial ubiquitin ligase; MnSOD=Manganese superoxide dismutase; Gpx=Glutathione peroxidase; Prx=Peroxiredoxin; Mfn=Mitofusin; Drp=Dynamin-related protein; Opa=Optic atrophy; Cox=Cytochrome C oxidase; Sdh=Succinate dehydrogenase; Pgc=PPARgamma coactivator; UCP=Uncoupling protein; HK=Hexokinase; PDK=Pyruvate dehydrogenase kinase; Fasn=Fatty acid synthase; SCD=Stearoyl-coenzyme A desaturase; SREBF=Sterol regulatory element binding transcription factor; Scap=SREBF cleavage-activating protein; Plin=Perilipin; HPRT=hypoxanthine-guanine phosphoribosyltransferase.
